# Loneliness and Social Engagement: The Unique Roles of State and Trait Loneliness for Daily Prosocial Behaviors

**DOI:** 10.1093/geroni/igaa057.2140

**Published:** 2020-12-16

**Authors:** Yeeun Lee, Jennifer Lay, Atiya Mahmood, Peter Graf, Christiane Hoppmann

**Affiliations:** 1 University of British Columbia, Vancouver, British Columbia, Canada; 2 Chinese University of Hong Kong, Hong Kong, China; 3 Simon Fraser University, Vancouver, British Columbia, Canada

## Abstract

Loneliness is a distressing yet adaptive emotional experience that alerts us to socially re-engage. However, loneliness can also lead to social withdrawal and isolation. To reconcile the seemingly contradictory consequences of loneliness, we unpack the timing of the underlying processes by distinguishing between the roles of state loneliness (i.e., daily variations in loneliness) and trait loneliness (i.e., person-average loneliness) in predicting social re-engagement. Using ten days of electronic daily assessments from 95 older adults (M age = 67.0 years; 64.2% women), initial findings indicate that trait loneliness moderates time-varying associations between state loneliness and prosocial behavior: On days of elevated state loneliness, older adults low in trait loneliness report increases in prosocial behavior, whereas older adults high in trait loneliness show decreases in prosocial behavior. Findings suggest that transient loneliness may motivate older adults to actively re-engage with others; chronic loneliness may undermine such adaptive responses.

